# Long-term clinical sequelae among Sudan ebolavirus disease survivors 2 years post-infection: a matched cohort study

**DOI:** 10.1186/s12916-025-04271-z

**Published:** 2025-07-18

**Authors:** Haruna Muwonge, Carolyne Nasimiyu, Barnabas Bakamutumaho, Peter Elyanu, Moses L. Joloba, Silvia Situma, John Schieffelin, Bronwyn Gunn, Shuangyi Bai, Robert F. Breiman, Isaac Ssewanyana, Susan Nabadda, Julius Lutwama, Yonas Tegen, Allan Muruta, Bruce Kirenga, Charles Olaro, Jane Ruth Aceng, Henry Kyobe Bosa, M. Kariuki Njenga

**Affiliations:** 1 Makerere University Medical School, Kampala, Uganda; 2 Center for Research in Emerging Infectious Diseases-East and Central Africa, Washington State University Global Health-Kenya, Nairobi, Kenya; 3 Uganda Virus Research Institute, Entebbe, Uganda; 4 Baylor College of Medicine Children Foundation-Uganda, Kampala, Uganda; 5 West Africa Research Network of Infectious Disease, Tulane University, New Orleans, USA; 6 Paul G. Allen School for Global Health, Washington State University, Pullman, USA; 7 Emory University, Atlanta, GA USA; 8 Uganda Central Public Health Laboratories, Kampala, Uganda; 9 Independent Global Health Consultant, Kampala, Uganda; 10 Ministry of Health Uganda, Kampala, Uganda

**Keywords:** Ebola virus disease, Sudan ebolavirus disease, Post-Ebola sequelae, Ebola survivors, Long-term health outcomes, Viral persistence, Reproductive health effects, Sub-Saharan Africa

## Abstract

**Background:**

The long-term health effects of ebolavirus disease (EVD) caused by the Sudan ebolavirus (SUDV) strain remain poorly characterized. Here, we assessed the nature, frequency, and persistence of post-EVD clinical symptoms among SUDV survivors 2 years after infection by comparing them with matched community controls.

**Methods:**

The primary objective was determining the prevalence of clinical symptoms over the 24-month period. Using a prospective matched cohort approach, 87 laboratory-confirmed SUDV survivors from the 2022–2023 Ugandan outbreak and 176 age-, sex- and village-matched controls were followed at 3, 9, 12, 15 and 24 months. Symptom data were collected through structured interviews and targeted clinical examinations. A secondary objective was investigating the duration of viral RNA shedding in semen and breast milk of survivors collected during follow-up, using the PCR test.

**Results:**

Of the 87 SUDV survivors, 57.5% reported significantly higher frequencies of clinical symptoms involving musculoskeletal (45.0%, *P* < 0.001), central nervous system (36.3%, *p* < 0.001), ophthalmologic (20%, *P* < 0.001), and respiratory (10%, *P* < 0.001) systems than those observed among controls. The risk ratio of occurrence was highest for ophthalmologic (20% vs 3.4%, RR = 5.9; *p* < 0.001) and central nervous systems symptoms (36.3% vs 6.8%, RR = 5.3, *p* < 0.001), and lowest for reproductive system (13.8% vs 8.5%; RR = 1.6; *p* > 0.005). Importantly, 50% of the survivors reported persistent multi-systemic symptoms, including low back pain, hand and feet numbness, confusion, and diarrhoea that resulted in an inability to perform basic activities of living. Viral RNA was detected in semen for up to 210 post-infection (median = 131 days, range: 111–210 days) and in breast milk for up to 199 days (median = 149 days, range: 111–199 days).

**Conclusions:**

This study demonstrates that SUDV survivors develop long-term clinical sequelae characterized by persistent multi-systemic clinical symptoms. Detection of viral RNA in semen and breastmilk for up to 7 months post-infection suggests prolonged persistence, opening the possibility of latency and reactivation of the virus.

**Supplementary Information:**

The online version contains supplementary material available at 10.1186/s12916-025-04271-z.

## Background

Ebolavirus disease (EVD) is a severe, often fatal haemorrhagic fever in humans that is often caused by four ebolavirus strains: Zaire (EBOV), Sudan (SUDV), Bundibugyo (BDBV), and Tai Forest (TAFV) viruses [1, 2]. While acute EVD has similar progression across the virus strains, with > 50% of cases developing life-threatening complications such as hypotension and acute multi-organ failure, the case fatality rate (CFR) ranges from 75 to 90% for EBOV, 55 to 65% for SUDV, and 25 to 33% for BDBV [2–4]. Over the last 30 years, EBOV and SUDV have been the most prevalent strains, responsible for > 70% of EVD epidemics, all originating from Africa [1, 2]. Recent studies, primarily in EBOV survivors, have described long-term EVD clinical sequelae characterized by sometimes severe and disabling musculoskeletal, neurological, psychological, ophthalmologic, and auditory symptoms that manifest from as early as 35 days after the acute EVD and can persist for years [5–7].

Following the 2014–2016 West African EBOV epidemic, studies identified a wide spectrum of long-term clinical sequelae, primarily with musculoskeletal and ophthalmologic symptoms [7–12]. Sustained mental health impacts included post-traumatic stress disorder, depression, substance abuse, anxiety, psychosis, and suicidal ideation [13, 14]. Ocular symptoms were associated with high viral load and severe acute EVD disease characterized by hemorrhagic manifestations [15]. More recent studies demonstrated that host-specific anti-EBOV immune responses correlated with clinical sequelae in part by predisposing individuals to virus persistence in immune-privileged sites, eliciting inflammatory reactions that yielded long-term clinical manifestations [16]. To investigate EVD clinical sequelae in survivors of SUDV strain, we enrolled 87 survivors from the 2022 outbreak in Uganda [17] shortly after discharge from Ebola treatment units (ETUs) and implemented long-term follow-up procedures. The outbreak, which started in September 2022, ended in January 2023, resulted in 142 confirmed SUDV cases, 55 deaths (CFR = 38.7%).

## Methods

### Study design and location

We conducted a prospective matched cohort study starting from February 2023 following SUDV survivors of the September 22–November 30, 2022, outbreak in Uganda. All 87 laboratory-confirmed SUDV survivors were consented and enrolled between February 1 and March 15, 2023, following discharge from Ebola Treatment Units (ETUs), a period regarded as 3 months post-infection. Using this timeline, subsequent follow-ups were conducted at 9, 12, 15 and 24 months post-infection, with the last 24-month follow-up completed on October 17, 2024. The study also enrolled 192 age-, sex- and location-matched community controls, drawn from the same or nearby villages as survivors. Controls were enrolled at a 2:1 ratio, with an additional 10% over-enrolment to account for potential seropositivity to SUDV. Seropositive controls (based on IgG reactivity to recombinant SUDV antigens) were subsequently excluded from analysis.

**Fig. 1:**
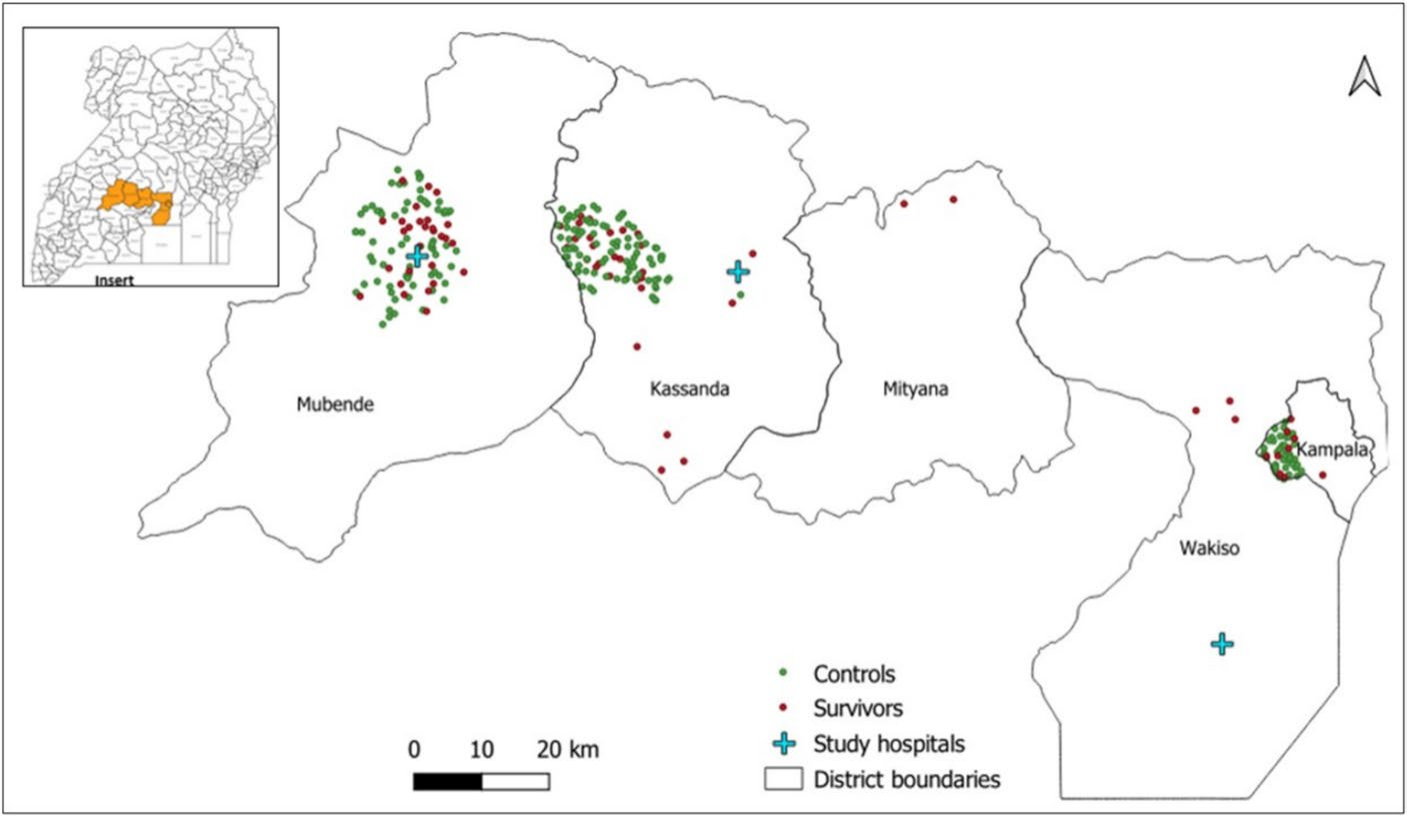
Spatial distribution of Sudan ebolavirus (SUDV) survivors and controls in Uganda**.** Map illustrating spatial distribution of SUDV survivors (red dots) and controls (green dots) across Mubende, Kassanda, Mityana, Wakiso, and Kampala districts. The study hospitals (blue crosses) include Mubende Regional Referral Hospital, Kikandwa Health Center III in Kassanda, and Entebbe Regional Referral Hospital in Wakiso. The insert map of Uganda shows the location of affected districts (orange shading)

Participant follow-up was embedded within an ongoing clinical and psychosocial support programme conducted by the MOH at three Uganda Ministry of Health (MOH) designated SUDV survivor clinics: Mubende Regional Referral Hospital, Kikandwa Health Centre III in Kassanda District, and Entebbe Regional Referral Hospital (Fig. 1). These health facilities are in the 3 districts that served as epicentres of the outbreak: Mubende, Kassanda, and Kampala districts.

### Participant enrolment

All 87-laboratory confirmed SUDV survivors were enrolled shortly after discharge from the Ebola Treatment Units (ETUs). Location, age, and sex-matched community controls were recruited from five villages where 86% of the survivors lived, with the number of controls proportional to the survivor population in each village. The matching criteria included location (same or nearby village), sex, and age (± 1 year for children < 5 years old, ± 2 years for participants 5–40 years old, and ± 5 years for those > 40 years). To minimize the chance of recruiting controls previously exposed to EVD, persons living in the same households with survivors, having a history of EVD exposure or fever between September and November 2022 were excluded.

### Clinical data collection and sampling

Data were collected at enrolment and during follow-up visits using structured questionnaires, medical file abstraction, and physical examinations. For survivors, detailed information was gathered regarding their EVD illness, including clinical presentation, management, past medical history, known EVD exposure risk factors, persistent symptoms after discharge from the ETUs, and reproductive health history. Follow-up visits focused on updating the participants’ current health status, capturing new symptoms, hospitalizations, exacerbations of existing conditions, and changes in sexual health. The questionnaires systematically collected symptom information, categorized by body system as summarized in Table 1.

**Table 1:** Symptom information collected during study visits, aggregated by body systems

Body system	Symptoms
General	Fever, headache, fatigue, weakness, anorexia, weight loss, rash
Musculoskeletal	Joint pain, limb numbness, muscular pain, back pain, joint swelling
Gastro-intestinal	Abdominal pain, diarrhoea, vomiting, nausea, difficulty swallowing
Respiratory	Sore throat, difficulty breathing, chest pain, cough
Ophthalmological	Blurry vision, eye pain, watery eyes, vision loss, dry eyes
Reproductive	Reduced libido, testicular pain, erectile dysfunction, menstrual period change
Central nervous system (CNS)	Memory loss, depression, confusion, difficulty in sleeping
Ear, nose, throat (ENT)	Tinnitus, hearing loss

Additional information regarding clinical signs, medical complications, laboratory test results, imaging findings, and treatments administered during ETU admission was abstracted from health records. Questionnaires were administered by trained study nurses, while additional targeted physical examinations were performed by a trained medical officer using the standard clinical methodology [18].

Venous blood was collected from controls, serum harvested, and transported to Uganda Central Public Health Laboratory to test for the presence of anti-SUDV antibodies and thus expulsion from the control group. From pregnant women, placental blood, amniotic fluid, and breast milk were collected, while baby tears and eye swabs were collected at delivery.

### Collection and testing semen and breast milk for viral RNA

During each visit, eligible adult male survivors provided semen while lactating females provided breast milk samples through self-expression to be tested for viral RNA. Specimen collection containers and instructions were provided by a trained Ministry of Health team involved in the psychosocial follow-up program. The specimens were triple packaged in accordance with international standards, refrigerated and transported to either the EVD mobile laboratory at Mubende Regional Referral Hospital or the enhanced biosafety level-2 laboratory at the Uganda Virus Research Institute (UVRI). At the laboratory, samples were placed into a negative pressure isolating glovebox and chemically inactivated using a mixture of virus lysis buffer and ethanol according to the manufacturer’s instructions (QIAmp viral RNA kit, Qiagen, Germany). Viral RNA was extracted from a 140-µl sample using the QIAmp viral RNA kit (Qiagen, Germany) followed by amplification cycling using the BioPerfectus ebolavirus real-time PCR kit (Jiangsu BioPerfectus Technologies, Taizhou, China) that detects both ebolavirus Zaire (EBOV) and SUDV strains.

### Screening sera from control participants for SUDV exposure

Sera samples collected from the 192 controls were assayed for IgG reactivity to recombinant SUDV glycoprotein (GP) and nucleoprotein (NP). Recombinant SUDV GP (IBT Bioservices, Cat# 0502-015) and NP (Sino Biologics, Cat# 40444-V07E1) were individually coupled to MagPlex (Luminex) magnetic beads with distinct fluorescent properties. Serum samples were diluted 100-, 500- and 1000-fold and incubated with the antigen-coated beads for 2 h at room temperature at 900 RPM. After washing, the beads were incubated with 0.65 µg/ml of PE-labelled secondary antibodies to detect IgG (total IgG, Southern Biotech, Cat# 2040-09) and analysed on IntelliFLEX SE-DR instrument (Luminex). Seropositivity threshold was determined using sera from 24 presumed SUDV-naïve controls (16 regional controls from Kenya and 8 controls from USA). For each antigen, we evaluated whether the data met the assumptions of normality. For the total IgG response to SUDV GP, the data followed a normal distribution as confirmed by the Shapiro–Wilk test. For the NP data, one outlier was identified using the Robust Regression and Outlier Removal (ROUT) method. After removing the outlier, the remaining samples followed a normal distribution pattern.

To minimize variability associated with a small sample size and maintain a conservative threshold, the seropositivity cut-off was calculated as mean plus five standard deviations (mean + 5 × SD) at each dilution level [19]. Control participants were classified seropositive if their mean fluorescence index exceeded that of SUDV GP at all three dilutions or at both the 500- and 1000-fold dilutions. Seropositive controls were excluded from the study.

### Data analysis

Clinical sequalae data cleaning and analysis were performed using R statistical software version 4.2.3. Descriptive analysis was conducted for categorical and numerical data and presented as frequencies, proportions, means and medians. Pearson’s chi-square test or Fisher’s exact test (where appropriate) was used to assess differences in symptom prevalence and other categorical variables between survivors and controls. Risk ratios (RRs) with 95% confidence intervals (CIs) were calculated to compare the likelihood of developing clinical sequelae among survivors relative to controls. For the current analysis, symptom prevalence was calculated based on data collected at the final follow-up visit, 24 months post-infection. These frequencies reflect symptoms present at that specific time point and do not represent cumulative prevalence across earlier visits. This approach was applied to both aggregated body system symptoms and specific symptom categories. For numerical variables, Student’s t-test or Mann–Whitney U test was used depending on data distribution, with statistical significance set at *p* ≤ 0.05. To understand the evolution of clinical sequelae over time, a temporal analysis was conducted, and the trends presented in the form of a heat map.

To assess the duration of viral RNA shedding in semen and breastmilk specimens, we recorded the number of days post-infection that participants remained PCR positive. The endpoint was defined as the last PCR positive result that was followed by two consecutive PCR negative results during follow-up.

### Ethical considerations

The study protocol was reviewed and approved by the School of Biomedical Sciences Research Ethics Committee at Makerere University (approval # SBS-2022-243) and the Uganda National Council of Science and Technology (approval # HS2618ES). Reliance was provided by Washington State University. Additional administrative approvals were sought from the Ministry of Health Uganda and the study health facilities. Written informed consents were obtained from all study participants. Those who declined were excluded from the study.

## Results

### Sociodemographic characteristics of Uganda SUDV survivors

The mean age of survivors was 31 (± 14) years while that of controls was 30 (± 13) years (Table 2). There was no significant difference in education level, occupation, or underlying conditions between survivors and controls.

**Table 2:** Sociodemographic characteristics of SUDV survivors and controls at time of enrolment

Characteristic	SUDV survivors, ***N*** = 87 *n* (%)	Controls, ***N*** = 176 *n* (%)	***p***-values
Sex			0.7
Male	56 (64.4)	109 (62)	
Age			> 0.9
Mean (SD)	31 (14)	30 (13)	
Median (IQR)	30 (23,38)	29 (23,38)	
Age groups			> 0.9
0–9	6 (6.9)	14 (8.0)	
10–19	11 (12.6)	19 (11)	
20–29	30 (34.5)	60 (34)	
30–39	25 (28.7)	50 (28)	
40–49	10 (11.5)	19 (11)	
50 +	5 (5.7)	14 (8.0)	
Level of education			> 0.9
None	45 (51.8)	82 (46.6)	
Primary	16 (18.4)	39 (22.2)	
Secondary	15 (17.2)	33 (18.7)	
Tertiary	11 (12.6)	22 (12.5)	
Main occupation			0.3
Farmer	31 (48.2)	66 (37.5)	
None	17 (19.5)	32 (18.2)	
Healthcare worker	8 (9.2)	3 (1.7)	
Skilled labour^a^	7 (8.0)	17 (9.7)	
Unskilled labour	5 (5.7)	4 (2.3)	
Other^b^	19 (21.8)	54 (30.7)	
Chronic conditions			NA
Hypertension	6 (6.9)	7 (3.6)	
HIV	5 (5.7)	6 (3.4)	
Diabetes	2 (2.3)	1 (0.5)	
Others	6 (6.9)	2 (1.1)	

^a^Except healthcare workers

^b^Businessmen, hairdressers, chefs, carpenters, shop attendants

### Comparing systemic clinical symptoms among survivors and controls

**Fig. 2:**
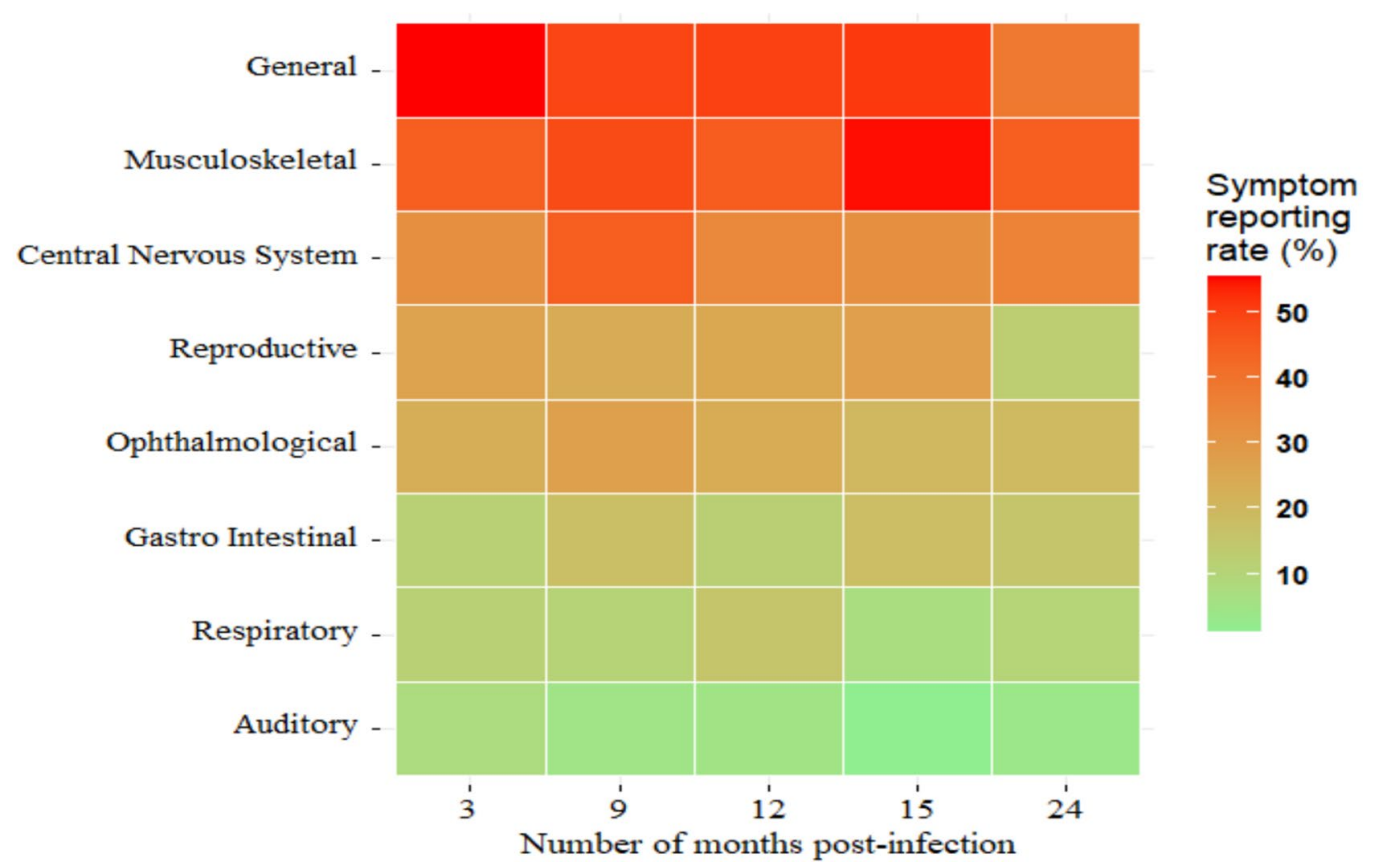
Heat map showing symptom reporting among Sudan ebolavirus (SUDV) survivors over 24 months. This heat map illustrates the proportion of SUDV survivors reporting symptoms across different body systems at 3, 9, 12, 15, and 24 months post-infection. The colour gradient represents the symptom reporting rate (%), with red shades indicating higher prevalence (> 50%) and green shades representing lower prevalence (< 10%)

Overall, 57.5% (50 of 87) of SUDV survivors developed at least one clinical symptom during the follow-up period, when compared to 32.4% of controls (RR = 1.8, *p* < 0.001). When compared to controls, there was a significantly higher frequency of clinical symptoms among survivors for the musculoskeletal, central nervous system (CNS), ophthalmological, reproductive, respiratory, and gastrointestinal systems (*P* < 0.001, Table 3, Fig. 2). The risk ratios were highest for ophthalmologic and CNS symptoms among SUDV survivors: 5.9 (20% vs 3.4%, *P* < 0.001) and 5.3 (36.3% vs 6.8%, *P* < 0.001), respectively, when compared to controls, and lowest in reproductive system symptoms was < 2 (Table 3).

**Table 3:** Frequency of systemic clinical symptoms among SUDV survivors and controls at 24 months post-infection

Body system	Survivors, *N* = 80 *n* (%)	Controls, *N* = 176 *n* (%)	Risk ratio	95% CI	*p*-value
Overall	46 (57.5)	57 (32.4)	1.8	1.3, 2.4	< 0.001
Musculoskeletal^a^	36 (45.0)	25 (14.0)	3.2	2.1,4.9	< 0.001
General^b^	31 (38.8)	34 (19.0)	2.0	1.3,3.0	0.002
Central nervous system^c^	29 (36.3)	12 (6.8)	5.3	2.9,9.9	< 0.001
Ophthalmological^d^	16 (20.0)	6 (3.4)	5.9	2.4,14.4	< 0.001
Reproductive^e^	11 (13.8)	15 (8.5)	1.6	0.8,3.4	0.026
Respiratory^f^	8 (10.0)	4 (2.3)	4.4	1.4,14.2	< 0.001
Gastro-intestinal^g^	12 (15.0)	7 (4.0)	3.8	1.5,9.2	0.011
Auditory^h^	3 (3.8)	1 (0.6)	6.6	0.7,62.5	0.092

^a^Muscle pain, joint pain, hand and feet numbness, lower back pain

^b^Fever, fatigue, general body weakness, headache, weight loss, anorexia

^c^Stiff neck, confusion, seizures, difficulty sleeping

^d^Eye pain, dry eyes, watery eyes, blurry vision, vision loss

^e^Irregular menstrual periods, erectile dysfunction, testicular pain, reduced libido

^f^Difficulty breathing, chest pain, sore throat, depression

^g^Abdominal pain, diarrhoea, nausea, vomiting

^h^Hearing loss, buzzing sound in the ears

Overall, symptoms related to the musculoskeletal (45%) system were the most frequent symptoms observed among survivors, although these were detected more often in controls as well (Table 3). The number of survivors reporting clinical symptoms was stable over time: 55.2% (48 of 87) at enrolment and 57.5% (46 of 80) at 2 years post-infection. Clinical symptoms associated with general, musculoskeletal, CNS, and ophthalmological systems were sustained at comparable proportions at 3, 9, 12, and 24 months post-infection (Fig. 2).

### Characterization of specific clinical symptoms

As shown in Table 4, the most frequent specific clinical symptoms reported by survivors were memory loss (35.0%), lower back pain (31.3%), hand and feet numbness (25.0%), and headache (21.3%). When compared to controls, the risk ratios of reported memory loss, blurry vision, depression, and sore throat were > 5.5 among survivors, and for reporting joint pains, weakness, eye pain, chest pain, hand and feet numbness, headache, and muscular pain, the risk ratios were between 3 and 5.5 (Table 4).

**Table 4:** Frequency and relative risk of specific clinical symptoms reported by SUDV survivors and controls at 24 months post-infection

Symptoms	Survivors, *N* = 80 *n* (%)	Controls, *N* = 176 *n* (%)	Risk ratio	95% CI	*P*-values
Memory loss	28 (35.0)	9 (5.1)	6.8	3.4,13.8	< 0.001
Lower back pain	25 (31.3)	19 (10.8)	2.9	1.7,4.9	< 0.001
Hand feet Numbness	20 (25.0)	13 (7.4)	3.4	1.8,6.5	< 0.001
Headache	17 (21.3)	11 (6.3)	3.4	1.7,6.9	0.001
Weakness	16 (20.0)	7 (4)	5.0	2.2,11.7	< 0.001
Joint pain	12 (15.0)	5 (2.8)	5.3	1.9,14.5	0.001
Fatigue	10 (12.5)	15 (8.5)	1.5	0.7,3.1	0.365
Blurry vision	9 (11.3)	3 (1.7)	6.6	1.8,23.7	0.002
Chest pain	7 (8.8)	4 (2.3)	3.9	1.2,12.2	0.039
Eye pain	6 (7.5)	3 (1.7)	4.4	1.1,17.2	0.029
Muscular pain	6 (7.5)	4 (2.3)	3.3	1.0,11.4	0.075
Depression	5 (6.3)	2 (1.1)	5.5	1.1,27.8	0.032
Weight loss	4 (5.0)	5 (2.8)	1.8	0.5,6.4	0.467
Anorexia	3 (3.8)	4 (2.3)	1.7	0.4,7.2	0.681
Sore throat	3 (3.8)	1 (0.6)	6.6	0.7,62.5	0.092

Of the 46 (57.5%) survivors that reported at least one symptom at 2 years post-infection time point, 60.9% reported more than three symptoms, 26.1% reported 2–3 symptoms, and 13% reported one symptom. Five of the 46 (10.9%) symptomatic participants reported symptoms that interfered with their daily activities, including hand and feet numbness, lower back pain, reduced libido, confusion, and diarrhoea. Overall, 50% (40/80) of all SUDV survivors reported persistent multiple (> 2) symptoms over the 2-year follow-up period.

### Age and gender differences in symptom presentation

SUDV survivor demographics played a significant role in long-term clinical sequelae. Older survivors reported joint pain significantly more frequently than younger age groups (*χ*^2^ = 16.87, *p* = 0.005). Although other symptoms such as memory loss, lower back pain, and hand/feet numbness were reported across age groups, these differences were not statistically significant. Symptom distribution across different age groups is shown in Additional file 1: Table S1.

Gender also influenced symptom presentation among survivors. Female survivors more commonly reported fatigue (RR = 0.24, *p* = 0.018), weakness (RR = 0.34, *p* = 0.015), headache (RR = 0.40, *p* = 0.029), and depression (RR = 0.14, *p* = 0.036) than their male counterparts. These differences highlight potential sex-specific vulnerabilities in post-SUDV sequelae. Additional file 2: Table S2 presents the frequency and relative risk of specific clinical symptoms stratified by gender among SUDV survivors.

### Detection of viral RNA in semen and breastmilk for over 6 months

**Fig. 3:**
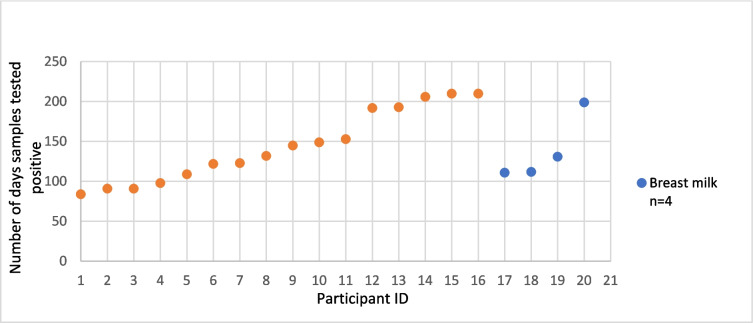
Duration of Sudan ebolavirus (SUDV) RNA detection in semen and breast milk samples. This figure illustrates the number of days that semen (orange, *n* = 16) and breast milk (blue, *n* = 4) samples tested positive for SUDV RNA by PCR. Each dot represents an individual participant

Of 47 male SUDV survivors aged ≥ 16 years and eligible for semen testing, 33 (70.2%) consented and were tested at enrolment, where SUDV RNA was detected in 16 (48.5%) by PCR. Four lactating mothers consented to breastmilk testing, and all were PCR positive for SUDV RNA at enrolment. Sixteen (*N* = 16) viral RNA positive men and the 4 lactating mothers agreed to subsequent testing during monthly psychosocial follow-up visits in accordance with World Health Organization guidelines until two consecutive samples were PCR negative. As shown in Fig. 3, the median duration for semen positivity was 131 days, with the longest duration being 210 days. For breast milk, the median duration of PCR positivity was 149 days, with the longest duration of positivity being 199 days.

### Survivor pregnancy outcomes

Four out of five (80%) survivors who became pregnant during the study period delivered babies at term without complications or abnormalities. One (20%) survivor reported spontaneous first-trimester abortion at 12 weeks’ gestation. All post-partum samples, including amniotic fluid, placental cord blood, breastmilk, baby tears and conjunctival swabs, tested negative for SUDV RNA by PCR.

## Discussion

This study characterized, for the first time, the type and prevalence of clinical symptoms observed during long-term EVD sequelae associated with SUDV, in a case-control approach that enhanced interpretation of findings. At 2 years, statistically significant symptom-specific risk ratios ranged between 2.9 and 6.8 when compared to controls. Memory loss, lower back pain, hand and feet numbness, and headache were the most prevalent symptoms among survivors. Importantly, symptoms were sustained at comparable levels across the 2-year follow-up period, consistent with studies in EBOV survivors which reported sustained clinical symptoms over the 4 years after infection [5]. Over longer periods of time, studies suggest that symptoms appear to resolve for EBOV and BDBV survivors; several studies note higher prevalence of clinical symptoms during convalescent periods when compared to 5 years later [8, 20, 21] This finding remains to be demonstrated in SUDV survivors. In our study, a substantial proportion of survivors reported debilitating symptoms, such as lower back pain, hand and feet numbness, reduced libido, confusion, and diarrhoea that interfered with wellness and daily living, such as working in subsistence farming, cooking, and house cleaning.

Some of the high-risk symptoms reported among SUDV survivors were consistent with those reported among EBOV strain survivors, including high prevalence of musculoskeletal and ophthalmological clinical symptoms [6, 22]. We found 26.5% and 14.5% of SUDV survivors reporting arthralgia and muscular pain, respectively, compared to 18–87% and 43% among EBOV survivors [22–24]. However, the prevalence of some clinical symptoms was different between EBOV and SUDV survivors. For example, while ear, nose, and throat (ENT) symptoms were among the least prevalent (4.8%) in SUDV survivors, a > fivefold (27%) higher proportion of EBOV survivors reported tinnitus and hearing loss [7, 23]. On the other hand, we found a higher prevalence of CNS symptoms including memory loss (35%), hand and feet numbness (25%), and depression (5%) than those reported for EBOV survivors (12% memory loss, 12% hand numbness, 16% feet numbness) [25–27]. Whether these differences reflect different viral pathogenesis pathways or differential host immune responses to the virus strains merits further investigations.

Although the pathophysiological mechanisms for long-term clinical sequelae following EVD remain poorly understood, they are speculated to involve either (i) direct virus pathways by persisting in tissues such as CNS and eye [28], (ii) host immune activation leading to inflammation and tissue damage or autoantibodies-mediated pathophysiology [29], or (iii) both pathways. The ebolavirus ability to evade the immune system and establish latency has been demonstrated [30] and an association between higher viral load during acute EVD and the development of certain sequelae is described [31]; the latter suggesting that the initial severity of the infection may influence the likelihood and severity of chronic EVD sequelae.

In our study, there was evidence of persistent virus infection in male and lactating female SUDV survivors, indicated by detection of SUDV RNA in semen and breastmilk for an average of 5 months, and for some men up to 2 years post-infection in semen. Importantly, in two males SUDV RNA was detected in semen 8 months after negative results on consecutive samplings, suggesting latency and reactivation of the virus. Similar findings have been reported for EBOV, documenting viral RNA presence for up to 18 months after infection [32, 33]. Detection of SUDV RNA in breastmilk signifies a notable risk for vertical transmission from mother to child, while virus in semen confirms the risk of sexual transmission as reported in some studies [33–38]. Despite the paucity of documented secondary transmission through these pathways, the potential warrants implementation of informed breastfeeding guidelines for SUDV survivors, and safe sex training for ebolavirus-discordant couples. Breastfeeding guidelines carefully balance the benefits of breastfeeding with the risks of ebolavirus transmission, optimizing the safety of both survivors and their infants while drawing on the latest research findings to provide optimal evidence-based recommendations [39].

SUDV survivor demographics played a significant role in long-term clinical sequelae, with older survivors reporting joint pain significantly more frequently (*χ*^2^ = 16.87, *p* = 0.005), while female survivors more commonly reported fatigue (RR = 0.24, *p* = 0.018), weakness (RR = 0.34, *p* = 0.015), headache (RR = 0.40, *p* = 0.029), and depression (RR = 0.14, *p* = 0.036). These findings mirror those observed in EBOV survivors [5, 40], underscoring distinct age- and gender-related patterns of post-EVD symptomatology. Taken together, these results highlight the need for tailored care strategies that address the unique needs of different demographic groups, as well as further research to refine management guidelines for EVD survivors.

While we found that 4 of the 5 (80%) female survivors who became pregnant after infection had normal birth outcomes, and that both maternal and foetal postpartum tissues were negative for viral RNA, the numbers are too small to conclude minimal risk of vertical transmission. However, studies among a larger cohort of EBOV survivors reported adverse pregnancy outcomes, including high rates of spontaneous abortion and stillbirths, that were reported among EBOV survivors [9, 41]. These findings suggest continued follow-up studies among pregnant EVD survivors before the development of policy guidelines regarding the impact of EVD on pregnancy outcomes.

Our study had some limitations. First, the small sample size and limited sampling timepoints precluded our ability to identify significant trends in sequelae. Second, the study was observational, with self-reporting as the primary source of clinical sequelae data, thus limiting causal inferences. Third, we did not collect detailed medical history or baseline symptom profiles for survivors prior to their acute SUDV infection, which limited our ability to adjust for pre-existing conditions as potential confounders in our comparisons with controls. This may have introduced some degree of selection bias in interpreting symptom differences. However, follow-up examination of survivors reporting certain symptoms by a trained medical officer strengthened the interpretation of the findings, as did the similarly collected data from matched controls. Data collected from future outbreaks would be helpful, as will continued follow-up of the cohort to continue characterizing long-term health impacts of SUDV.

## Conclusions

Our study demonstrates that SUDV survivors, much like EBOV survivors, develop long-term clinical sequelae characterized by persistent multi-systemic debilitating clinical symptoms. Our virology findings confirm SUDV persistence in survivors, with potential for latency and reactivation that may be a source of future human-to-human transmission and outbreaks.

## Supplementary Information

Additional File 1: Table S1: Distribution of specific symptoms by age group among SUDV survivors. This table presents the frequency of each clinical symptom stratified by age group. Statistical comparisons were made using chi-square tests to assess associations between symptom occurrence and age category.

Additional File 2: Table S2: Frequency and relative risk of specific clinical symptoms by gender among SUDV survivors. This table details the number of male and female survivors reporting each symptom and presents the associated risk ratios, 95% confidence intervals, and *p*-values.

## Data Availability

The datasets generated and/or analyzed during the current study are not publicly available due to participant confidentiality and institutional data protection policies, but are available from the corresponding author on reasonable request.

## Abbreviations

BDBV: Bundibugyo ebolavirus
CFR: Case fatality rate
CI: Confidence interval
CNS: Central nervous system
EBOV: Ebola virus (Zaire strain)
ENT: Ear, nose, and throat
ETU: Ebola treatment unit
EVD: Ebolavirus disease
GP: Glycoprotein
IgG: Immunoglobulin G
IQR: Interquartile range
MOH: Ministry of Health
NP: Nucleoprotein
PCR: Polymerase chain reaction
PE: Phycoerythrin
RNA: Ribo-nucleic acid
RR: Risk ratio
SD: Standard deviation
SUDV: Sudan ebolavirus
TAFV: Tai Forest ebolavirus
UVRI: Uganda Virus Research Institute
