# Supplementary material for: Long-term clinical sequelae among Sudan ebolavirus disease survivors 2 years post-infection: a matched cohort study
Source: BMC Med. 2025 Jul 18;23:432. doi: 10.1186/s12916-025-04271-z (PMC12275283; doi:10.1186/s12916-025-04271-z)
Supplement: Supplementary file 1 — Additional File 1: Table S1: Distribution of specific symptoms by age group among SUDV survivors. This table presents the frequency of each clinical symptom stratified by age group. Statistical comparisons were made using chi-square tests to assess associations between symptom occurrence and age category. [file 12916_2025_4271_MOESM1_ESM.docx]

**Table S1:** *Distribution of specific symptoms by age group among SUDV survivors*

| **Symptoms** | **N** | **Chi-Square** | **df** | **p-value** | **Age group (yrs)** | | | | | |
| --- | --- | --- | --- | --- | --- | --- | --- | --- | --- | --- |
|  |  |  |  |  | **0–9** | **10–19** | **20–29** | **30–39** | **40–49** | **50+** |
| Fatigue | 10 | 2.35 | 5 | 0.80 | NA | 1 | 4 | 3 | 2 | NA |
| Weakness | 16 | 5.02 | 5 | 0.41 | NA | 1 | 5 | 6 | 2 | 2 |
| Anorexia | 3 | 7.88 | 5 | 0.16 | NA | NA | 1 | NA | 1 | 1 |
| Weight Loss | 4 | 4.90 | 5 | 0.43 | NA | NA | 3 | NA | 1 | NA |
| Sore Throat | 3 | 3.94 | 5 | 0.56 | NA | 1 | NA | 1 | 1 | NA |
| Headache | 17 | 5.00 | 5 | 0.42 | 1 | NA | 7 | 5 | 2 | 2 |
| Memory Loss | 28 | 1.68 | 5 | 0.89 | 2 | 4 | 10 | 6 | 4 | 2 |
| Hand/Feet Numbness | 20 | 4.50 | 5 | 0.48 | NA | 2 | 6 | 7 | 4 | 1 |
| Chest Pain | 7 | 4.79 | 5 | 0.44 | NA | NA | 4 | 2 | NA | 1 |
| Depression | 5 | 3.12 | 5 | 0.68 | NA | NA | 3 | 2 | NA | NA |
| Muscular Pain | 6 | 3.28 | 5 | 0.66 | NA | NA | 2 | 2 | 1 | 1 |
| Joint Pain | 12 | 16.87 | 5 | 0.005 | 1 | NA | 2 | 3 | 3 | 3 |
| Lower Back Pain | 25 | 8.79 | 5 | 0.12 | NA | 1 | 13 | 6 | 3 | 2 |
| Eye Pain | 6 | 3.59 | 5 | 0.61 | 1 | 1 | 3 | NA | 1 | NA |
| Blurry Vision | 9 | 10.92 | 5 | 0.053 | 1 | NA | 5 | 1 | NA | 2 |
