# Supplementary material for: Long-term clinical sequelae among Sudan ebolavirus disease survivors 2 years post-infection: a matched cohort study
Source: BMC Med. 2025 Jul 18;23:432. doi: 10.1186/s12916-025-04271-z (PMC12275283; doi:10.1186/s12916-025-04271-z)
Supplement: Supplementary file 2 — Additional File 2: Table S2: Frequency and relative risk of specific clinical symptoms by gender among SUDV survivors. This table details the number of male and female survivors reporting each symptom and presents the associated risk ratios, 95% confidence intervals, and p-values. [file 12916_2025_4271_MOESM2_ESM.docx]

**Table S2:** *Frequency and relative risk of specific clinical symptoms by gender among SUDV survivors*

| **Symptoms** | **Male (N=51)** | **Female (N=29)** | **Risk Ratio** | **Confidence Interval** | **P-Value (χ²)** |
| --- | --- | --- | --- | --- | --- |
| Fatigue | 3 | 7 | 0.24 | 0.07 – 0.87 | 0.018* |
| Weakness | 6 | 10 | 0.34 | 0.14 – 0.84 | 0.015* |
| Anorexia | 0 | 3 | 0 | NA | 0.019* |
| Weight Loss | 2 | 2 | 0.57 | 0.08 – 3.83 | 0.557 |
| Sore Throat | 1 | 2 | 0.28 | 0.03 – 3.00 | 0.264 |
| Headache | 7 | 10 | 0.40 | 0.17 – 0.93 | 0.029* |
| Memory Loss | 14 | 14 | 0.57 | 0.32 – 1.02 | 0.060 |
| Hand/Feet Numbness | 8 | 12 | 0.38 | 0.18 – 0.82 | 0.011* |
| Chest Pain | 2 | 5 | 0.23 | 0.05 – 1.10 | 0.043* |
| Depression | 1 | 4 | 0.14 | 0.02 – 1.21 | 0.036* |
| Muscular Pain | 3 | 3 | 0.57 | 0.12 – 2.64 | 0.466 |
| Joint Pain | 7 | 5 | 0.80 | 0.28 – 2.28 | 0.672 |
| Lower Back Pain | 11 | 14 | 0.45 | 0.23 – 0.85 | 0.013* |
| Eye Pain | 5 | 1 | 2.84 | 0.35 – 23.17 | 0.299 |
| Blurry Vision | 6 | 3 | 1.14 | 0.31 – 4.21 | 0.847 |
